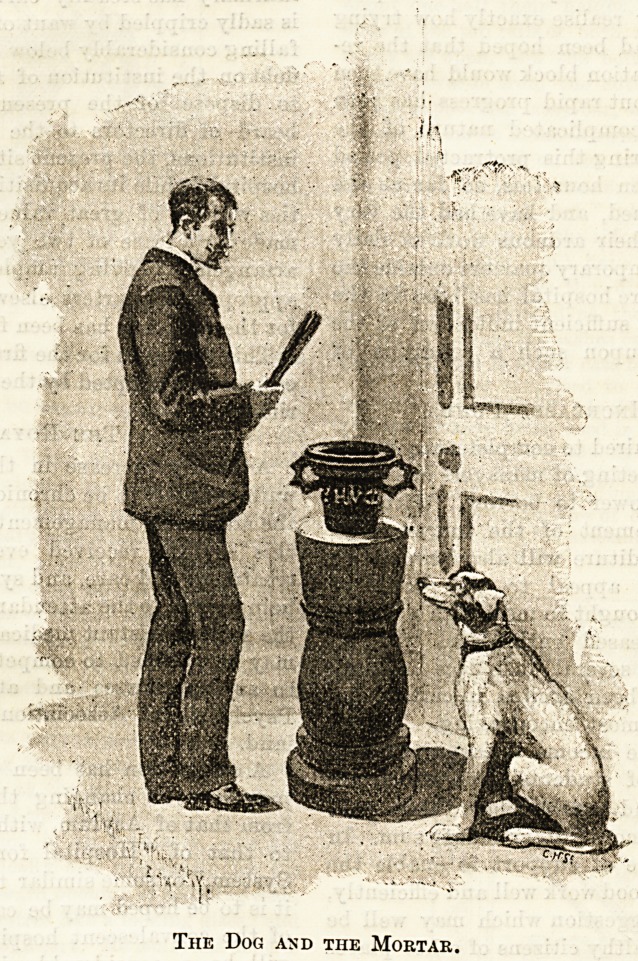# Wrexham Infirmary

**Published:** 1894-03-17

**Authors:** 


### The Institutional Workshop.

### WITHIN THE HOSPITALS.

### WREXHAM INFIRMARY.

(By a Vagrant Correspondent.)

Wrexham, or as it is sometimes called by its inhabitants, "The Little City of Spires," owing to the numerous churches which seem to cluster together in its centre, has an excellent infirmary. The building stands in Regent Street, and is only a few hundred yards from the railway station. On passing under its rather pretentious portico, which is after the style of that of the Stockport Infirmary, I found myself in a pleasant little hall, which seemed to be always open, and also served as a waiting-room. The only occupant of the hall for the moment was a fox terrier, who went by the rather questionable appellation of "Nick," as I discovered by the insignia round his neck. He wagged his tail as I entered, and at once seemed to act as showman by drawing my attention to the most interesting relic I discovered in the place, an old iron pestle and mortar, standing on a pedestal of oak about three feet from the ground. Inscribed on the mortar were the name of Hugh Meredith, and the date 1685. The dog then walked to the other corner of the hall, and drew my attention to an elaborate vase full of bulrushes, which his artistic instinct thought I might like to take note of. The walls were painted in light yellow, and a terra-cotta dado decorated their base. In red, black, and gold on white boards arranged on the walls were the names of many of the subscribers to the hospital fund, amongst whom those of Peter Walker, of Coed-y-glyn, and Miss Mary Trehearne, of Dublin, figured conspicuously for their munificent donations.

The Dog and the Mortar.

On ringing the bell of the inner hall, the dog augmented my call with a sharp bark, and I was soon admitted into the corridor. The main building, which is principally occupied by the staff, consists of two long upper and lower corridors, with rooms on either side. There is a children's ward fronting the street furnished with six cots. It is a very cheerful room, dadoed with blue tiles and walled with bright pictures. There were only three cots occupied, and the little tenants of these were having their dinner. If their appetites were any sign, the youngsters' troubles were not great. There were two other wards, containing six and four beds each, for isolated cases. Branching out from the main building at the back, somewhat after the pavilion system, were the principal wards, containing ten beds each. The upper men's ward opened by a large French window into a spacious balcony, and thence by iron stairway the patients could descend into the garden. The balcony was quite large enough for many garden chairs, and in the summer must be a great boon to the more convalescent of the sufferers. The operating theatre, general surgery, and the lavatories were excellent in every way in this establishment. The latest addition to the Wrexham Infirmary is a nurses' home. This is slightly apart from the main building; it contains a sitting-room, a bath, and three bed rooms, all decorated in a light, airy style, with tiled dadoes.

## Figures and Tables

**Figure f1:**